# Inhibition of CRY2 by STAT3/miRNA-7-5p Promotes Osteoblast Differentiation through Upregulation of CLOCK/BMAL1/P300 Expression

**DOI:** 10.1016/j.omtn.2019.12.020

**Published:** 2019-12-24

**Authors:** Zhenghui Tang, Tianyuan Xu, Yinghua Li, Wenchao Fei, Gong Yang, Yang Hong

**Affiliations:** 1 Central Laboratory, The Fifth People’s Hospital of Shanghai, Fudan University, Shanghai 200240, China; 2 Department of Orthopedics, The Fifth People’s Hospital of Shanghai Fudan University, Shanghai 200240, China; 3 School of Life Sciences, Shanghai University, Shanghai 200244, China; 4 Cancer Institute, Fudan University Shanghai Cancer Center, Shanghai, 200032, China; 5 Department of Oncology, Shanghai Medical College, Fudan University, Shanghai, 200032, China

**Keywords:** osteogenic differentiation, miR-7-5p, cryptochrome circadian regulator 2, STAT3, mesenchymal stem cells

## Abstract

Accumulating evidence indicates that cryptochrome circadian regulatory (CRY) proteins have emerged as crucial regulators of osteogenic differentiation. However, the associated mechanisms are quite elusive. In this study, we show that knockdown of CRY2 downregulated the expression of runt-related transcription factor 2 (Runx2), alkaline phosphatase (ALP), osteocalcin (OCN), and osteopontin (OPN) to facilitate osteoblast differentiation. Further study identified that CRY2 was directly targeted by microRNA (miR)-7-5p, which was highly induced during osteoblast differentiation. The expression of Runx2, ALP, collagen type I alpha 1 (Col1a1), and OCN was upregulated by overexpression of miR-7-5p and induction of osteoblast differentiation. Moreover, signal transducer and activator of transcription 3 (STAT3) transcriptionally activated miR-7-5p to significantly enhance the expression of above osteogenic marker genes and mineral formation. However, overexpression of CRY2 abolished the osteogenic differentiation induced by miR-7-5p overexpression. Silencing of CRY2 unraveled the binding of CRY2 with the circadian locomotor output cycles kaput (CLOCK)/brain and muscle ARNT-like 1 (BMAL1) complex to release CLOCK/BMAL1, which facilitated the binding of CLOCK/BMAL1 to the promoter region of the P300 E-box to stimulate the transcription of P300. P300 subsequently promoted the acetylation of histone 3 and the formation of a transcriptional complex with Runx2 to enhance osteogenesis. Taken together, our study revealed that CRY2 is repressed by STAT3/miR-7-5p to promote osteogenic differentiation through CLOCK/BMAL1/P300 signaling. The involved molecules may be potentially targeted for treatment of osteoporosis.

## Graphical Abstract

**Figure:**
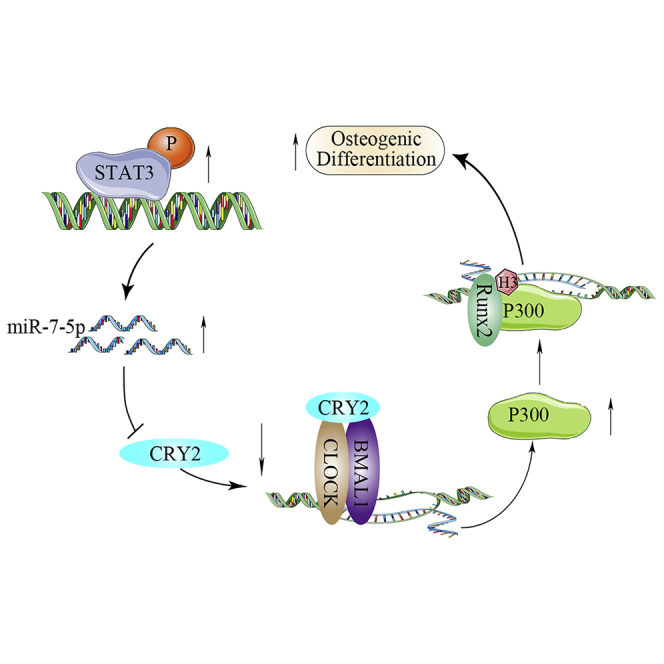

## Introduction

Osteoblast differentiation and bone remodeling are regulated by a concerted communication between osteoblasts and osteoclasts.^1^^,^^2^ Osteoblast differentiation requires numerous transcription factors to regulate both the formation and maintenance of bone.^3^ Osteoblasts differentiated from mesenchymal stem cells (MSCs) account for 5% of bone cells and are responsible for the synthesis of type I collagen and the deposition of the mineralized nodule to facilitate the formation of bone.^4^

CRY2, the cryptochrome circadian regulator 2, is a core component of the circadian clock necessary for the generation and maintenance of circadian rhythms.^5^ CRY2 acts as a transcriptional repressor to negatively regulate the gene transcription and is associated with diverse physiological processes.^6^ More and more reports suggest that the circadian rhythm may regulate specific osteogenic differentiation. For instance, animals with *Cry2*^−/^^−^ displayed a significant increase of the bone volume at the age of 12 weeks.^7^ Furthermore, the expression of peroxisome proliferator-activated receptor δ (PPARδ) genes was elevated in myotubes and muscles of the *Cry2*^−/−^ animal,^8^ which facilitates osteogenic differentiation through the sirtuin 1-dependent signaling pathway.^9^ However, the specific mechanism associated with CRY2 is unknown.

MicroRNAs (miRNAs) are short, noncoding RNAs that disturb the translation of specific target mRNAs to regulate various biological processes.^10^ An increasing number of miRNAs, such as miR-219a-5p, miR-23a, miR-451a, and miR-25, have been identified to promote osteoblast differentiation through targeting the negative regulators of osteogenesis.^3^^,^^11^, ^12^, ^13^, ^14^ In contrast, some other specific miRNAs promote the differentiation of osteoclasts by targeting critical osteogenic factors.^15^ For example, miR-221 proved to inhibit osteoblast differentiation by targeting the runt-related transcription factor 2.^16^ Furthermore, miRNA-376c-3p was identified to regulate the osteogenesis by targeting Twist-related protein 1 (TWIST-1) in bone marrow-derived MSCs (BMSCs).^17^ Similarly, miR-208a-3p targets Activin A receptor type I (ACVR1) to suppress osteoblast differentiation and bone formation.^18^ Numerous studies have demonstrated that miRNAs are indispensable for the progression of osteoblast or osteoclast differentiation.

In this study, we identified that the expression of CRY2 was directly inhibited by signal transducer and activator of transcription 3 (STAT3)-transactivated miR-7-5p overexpression, which upregulated the expression of circadian locomotor output cycles kaput (CLOCK)/brain and muscle ARNT-like 1 (BMAL1) and the transcription of P300 to stimulate osteogenesis through the enhanced histone 3 acetylation and the formation of a transcriptional complex with runt-related transcription factor 2 (Runx2). Successful identification of miR-7-5p might provide an alternative therapeutic strategy against CRY2 to maintain the bone anabolism in the context of age-related bone disease, such as osteoporosis.

## Results

### Knockdown of CRY2 Enhances Osteogenic Differentiation

**Figure 1:**
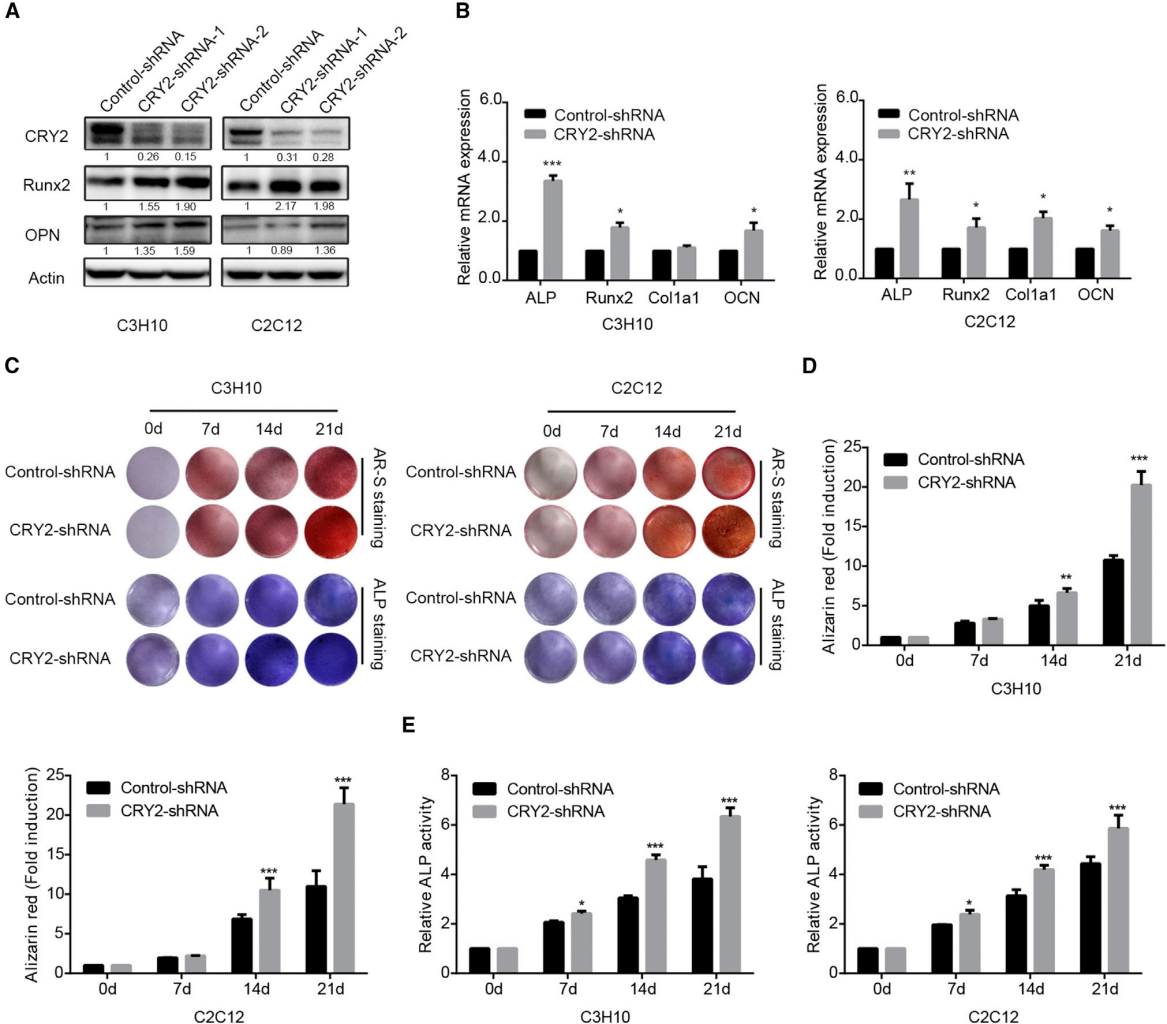
Knockdown of CRY2 Enhances Osteogenic Differentiation (A) Western blot analysis of CRY2 expression in shRNA stably transfected cells. (B) qRT-PCR was performed to determine the expression levels of OCN, ALP, Col1a1, and Runx2 in shRNA stably transfected cells and control cells. (C) Alizarin red staining and ALP staining in CRY2 shRNA stably transfected cells compared with control cells. (D) Alizarin red-S staining was quantified by densitometry at 562 nm. (E) Relative ALP activity was measured during osteoblastic differentiation of C3H10 and C2C12 cells. β-actin is a protein-loading control. All assays were repeated at least three times. The data are the mean ± SD (n = 3). n.s., not significant, *p < 0.05, **p < 0.01, ***p < 0.001.

To identify the role of CRY2, a transcriptional repressor and a core component of the circadian clock that may play a crucial role in osteogenic differentiation, we first silenced the endogenous expression of CRY2 in C3H10 and C2C12 cell lines by using a couple of short hairpins RNAs (shRNAs). The protein expression level of CRY2 was efficiently decreased by CRY2 shRNA compared with control shRNA (Figure 1A). Moreover, we used qRT-PCR to test the expression levels of osteogenic differentiation markers after the silencing of CRY2. The results showed that the expression of various bone-specific markers, including the runt-related transcription factor 2 (Runx2), alkaline phosphatase (ALP), the collagen type I alpha 1 (Col1a1), osteocalcin (OCN), and osteopontin (OPN), was upregulated after knockdown of CRY2 expression (Figure 1B). Furthermore, we examined the effect of CRY2 on osteogenic differentiation and mineralization. Cells expressing shRNA control and CRY2 shRNA were cultured in the osteogenic induction medium. After 21 days of osteogenic induction, mineralized nodules were stained red by Alizarin red staining, as shown in Figures 1C and 1D. Knockdown of CRY2 impaired the formation of mineralized nodules tested by Alizarin red staining. Similarly, ALP staining and ALP activity were significantly increased in CRY2 shRNA-treated cells compared with control cells (Figures 1C and 1E). These results suggest that CRY2 suppresses osteogenic differentiation.

### CRY2 Is a Direct Target of miR-7-5p

**Figure 2:**
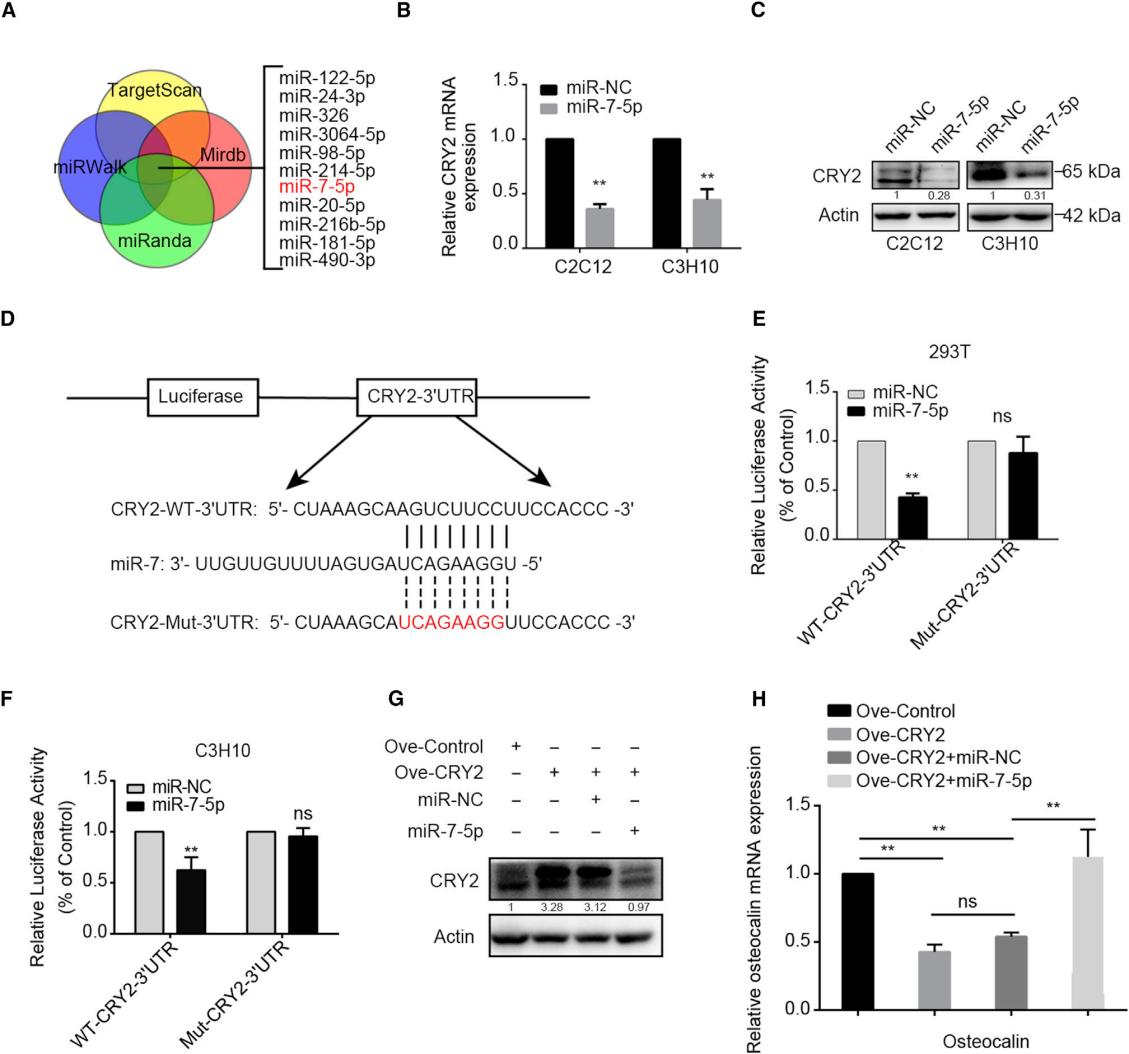
CRY2 Is a Direct Target of miR-7-5p (A) A diagram showing candidate miRNAs analyzed by bioinformatics. (B) The mRNA levels of CRY2 detected by qRT-PCR in miR-7-5p overexpression cells. (C) Western blot analyses of CRY2 protein expression in miR-7-5p overexpression cells compared with empty vector. (D) The putative binding sites of miR-7-5p to the 3′ UTR of CRY2 predicted by bioinformatic prediction tools, and the mutation site in the 3′ UTR of CRY2 was shown. (E and F) The effect of miR-7-5p on luciferase activity induced by the pGL3-CRY2-wt and pGL3-CRY2-mut reporter plasmids in HEK293T (E) and C3H10 (F) cells determined by luciferase reporter assays. (G) CRY2 expression in cells with miR-7-5p only or miR-7-5p plus CRY2 cDNA tested by western blot. (H) Quantification of OCN in cells transfected with miR-7-5p only or miR-7-5p plus CRY2 cDNA by qRT-PCR. β-actin is a protein-loading control. All assays were repeated at least three times. The data are the mean ± SD (n = 3). n.s., not significant. *p < 0.05, **p < 0.01, ***p < 0.001.

To investigate the molecular mechanism of the CRY2-associated osteogenic differentiation, we used TargetScan, miRWalk, microrna.org, and miRDB to predict the possible miRNAs that might target the 3′ UTR of CRY2 mRNA. Among the candidate miRNAs, we selected 11 miRNAs (Figure 2A), but we found that the expression of CRY2 was only decreased after miR-7-5p overexpression at the mRNA level (Figure 2B). Meanwhile, the protein level of CRY2 was downregulated in two cell lines (Figure 2C). Through the bioinformatic analysis, the wild-type (WT) and mutant 3′ UTR of CRY2 were cloned into the dual-luciferase reporter assay vector (Figure 2D). The luciferase reporter vector and pRL-TK vector (Renilla) reference vector were cotransfected along with miR-7-5p into HEK293T cells, and the luciferase activity was measured to determine the effects of miR-7-5p on luciferase expression. Overexpression of miR-7-5p significantly suppressed the luciferase activity in wild-type but not mutant CRY2 3′ UTR-transfected cells, whether exogenous or endogenous (Figures 2E and 2F). Furthermore, we found that overexpression of miR-7-5p could remarkably abolish the expression of CRY2 (Figure 2G). After CRY2 overexpression, the expression of osteocalcin was decreased. However, overexpression of miR-7-5p reversed the inhibition of osteogenic differentiation induced by CRY2 overexpression (Figure 2H). All data indicate that CRY2 may act as a downstream target of miR-7-5p.

### miR-7-5p Promotes Osteoblastic Differentiation

**Figure 3:**
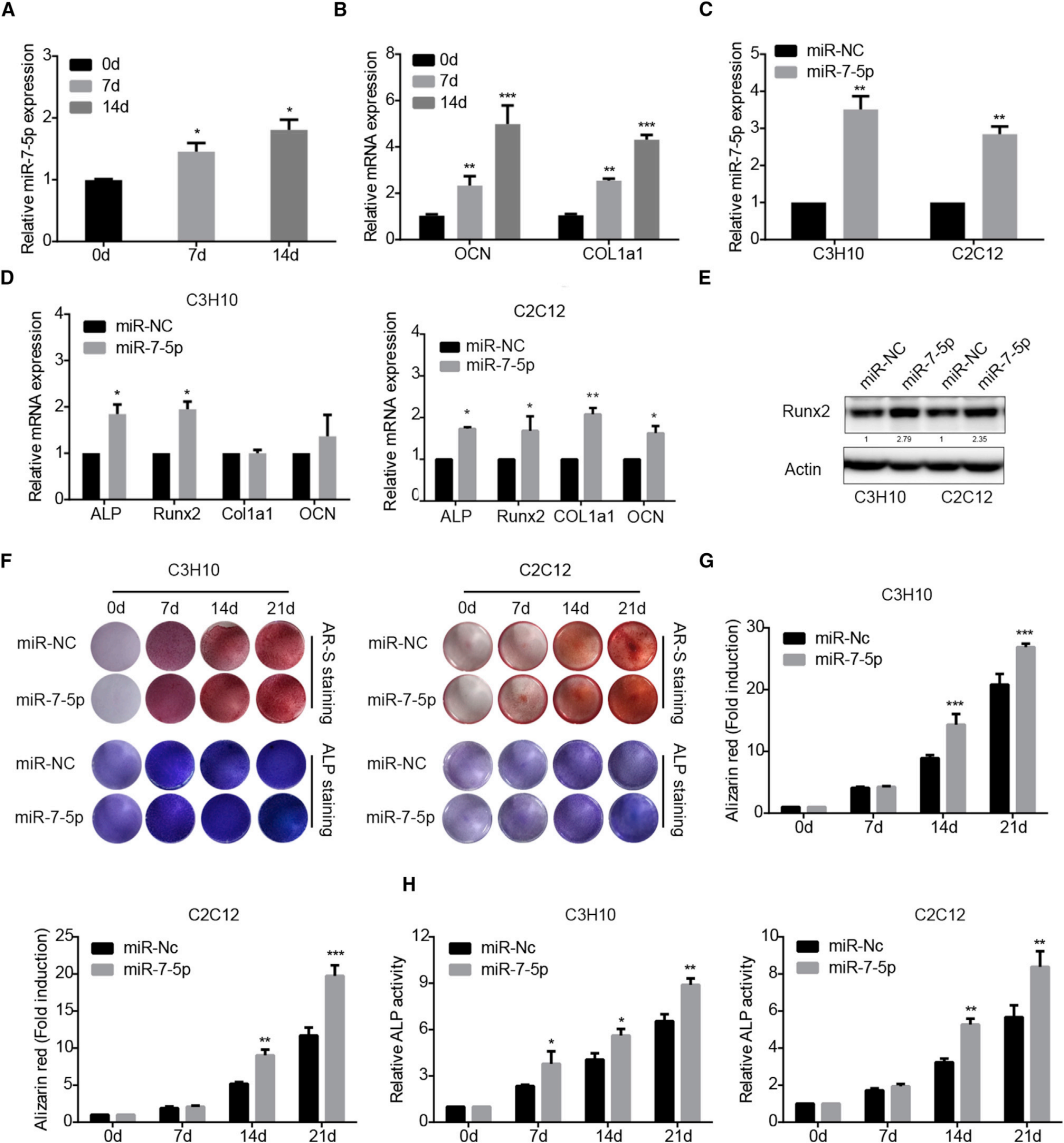
miR-7-5p Promotes Osteogenic Differentiation (A) Quantitative real-time PCR analysis of miR-7-5p expression during osteogenic differentiation. U6 was used as an internal control. (B) Quantitative real-time PCR analysis of Col1a1 and osteocalcin expression. 18S RNA was used as an internal control. (C) Relative miR-7-5p expression in miR-7-5p overexpression cells tested by qRT-PCR. (D) The expression levels of OCN, ALP, Col1a1, and Runx2 detected by qRT-PCR. (E) The expression of Runx2 in miR-7-5p overexpression cells detected by western blot. (F) Alizarin red staining and ALP staining in miR-7-5p overexpression cells compared with control cells. (G) Alizarin red-S staining was quantified by densitometry at 562 nm. (H) Relative ALP activity was measured during osteoblastic differentiation of C3H10 and C2C12 cells. β-actin is a protein-loading control. All assays were repeated at least three times. The data are the mean ± SD (n = 3). n.s., not significant, *p < 0.05, **p < 0.01, ***p < 0.001.

To detect the expression of miR-7-5p during osteoblastic differentiation, MSCs were treated with osteogenic induction medium. The expression levels of the osteogenic markers, including Col1a1 and OCN, were monitored on days 0, 7, and 14 upon the induction. We detected the expression of miR-7-5p by qRT-PCR during osteogenic differentiation and found that miR-7-5p expression was upregulated at the different time points of induction (Figure 3A). In addition, the relative mRNAs of Col1a1 and OCN were also upregulated (Figure 3B).

Based on above results, miR-7-5p was overexpressed by the lentivirus-mediated infection in mouse myoblast (C2C12) and mouse MSCs (C3H10). The results showed that the expression of miR-7-5p was remarkably increased (Figure 3C). Accordingly, overexpression of miR-7-5p could promote the expression of osteogenic gene markers, such as ALP, Runx2, Col1a1, and OCN (Figures 3D and 3E). The osteogenic differentiation of these cell lines was assayed by Alizarin red staining and ALP activity staining. We found that miR-7-5p overexpression promoted the formation of mineralized nodules and increased the ALP activity (Figures 3F–3H). These results suggest that miR-7-5p may participate in the process of osteogenic differentiation.

### miR-7-5p Is Transactivated by STAT3

**Figure 4:**
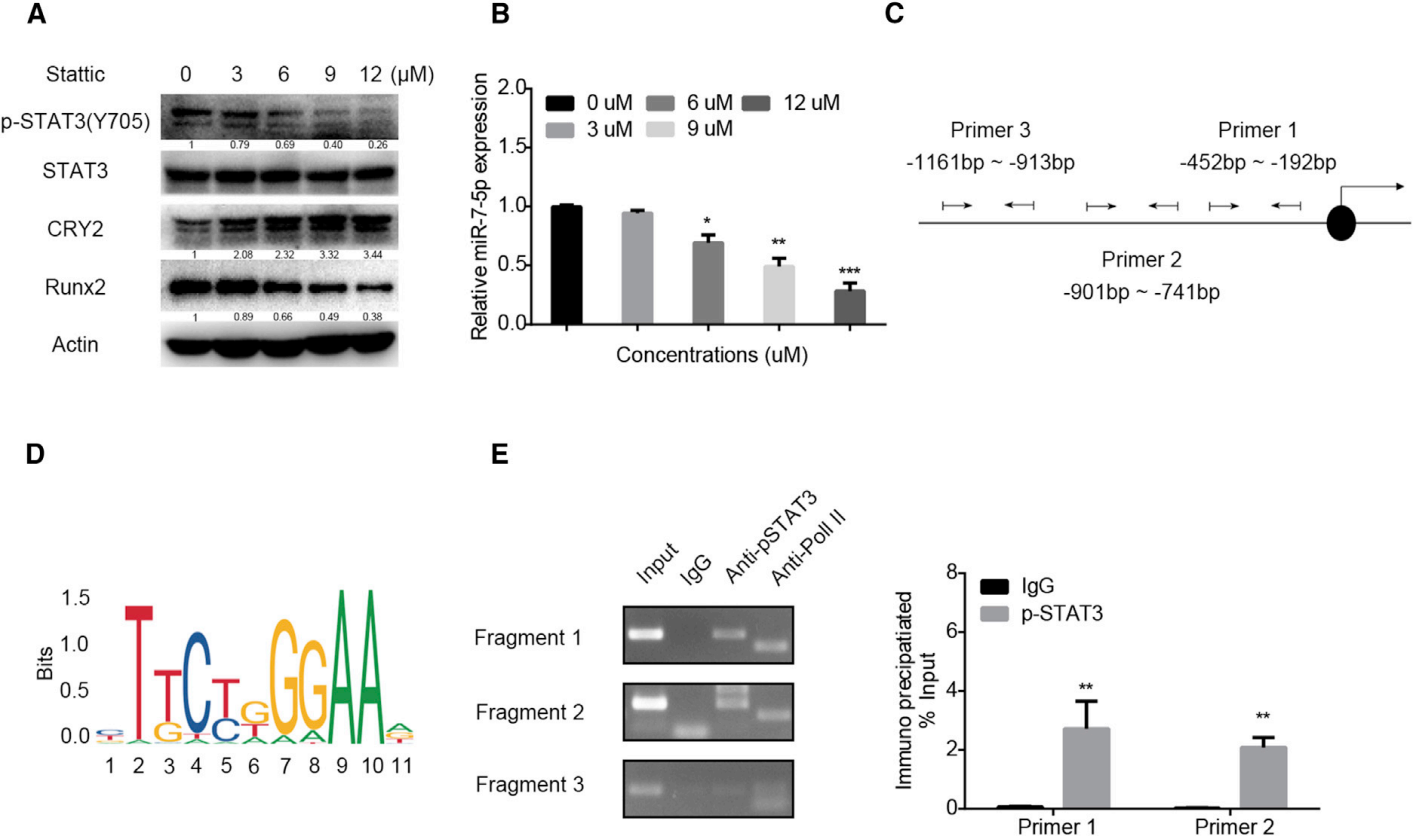
miR-7-5p Is Regulated by STAT3 (A) Western blot analysis of p-STAT3 (Y705), CRY2, and Runx2 expression in C3H10 cells after treatment with different concentrations of the STAT3 inhibitor Stattic. (B) qRT-PCR was performed to detect the expression levels of miR-7-5p after treatment with a different concentration of Stattic in C3H10 cells. (C) The promoter region of miR-7-5p containing the putative STAT binding sites. (D) Transcription start site (TSS) binding motives for STAT3 obtained from JASPAR. (E) Binding sites of STAT3 to the miR-7-5p promoter tested by chromatin immunoprecipitation (ChIP). IgG, negative control; Input, starting sonicated DNA; Pol II, RNA polymerase II positive control. β-actin is a protein-loading control. All assays were repeated at least three times. The data are the mean ± SD (n = 3). n.s., not significant, *p < 0.05, **p < 0.01, ***p < 0.001.

To identify further the transcriptional regulation of miR-7-5p during osteoblastic differentiation, we analyzed the sequence containing 3,000 nt located at the miR-7-5p promoter region by bioinformatics with online prediction databases (PROMO, Jaspar, UCSC Genome Browser, and hTFtarget). Three binding sites, including −342 bp to −358 bp (5′-CATTACAGGAACACAG-3′), −853 bp to −862 bp (5′-TACCAGGAA-3′), and −1,044 bp to −1,056 bp (5′-GGGTGGCTCCGG-3′), were identified to bind to STAT3. To test the effect of STAT3 on miR-7-5p expression, C3H10 cells were treated with the STAT3-specific inhibitor Stattic using different concentrations. western blot results showed that the expression level of phosphorylated (p)-STAT3 (Y705) was significantly suppressed, and CRY2, the target of miR-7-5p, was upregulated (Figure 4A) after treatment with Stattic. The miR-7-5p expression level was significantly decreased as well (Figure 4B). Next, we designed oligos from −452 bp to −192 bp, from −901 bp to −741 bp, and from −1,161 bp to −913 bp (Figures 4C and 4D) to test their binding activity to STAT3. By chromatin immunoprecipitation (ChIP) assay, we revealed that p-STAT3 (Y705) could directly bind to the promoter region of miR-7-5p to activate the transcriptional ability (Figure 4E). All of these results confirmed that STAT3 could positively activate the miR-7-5p expression.

### Knockdown of CRY2 Enhances Osteogenic Differentiation to Promote CLOCK/BMAL1/P300 Expression

**Figure 5:**
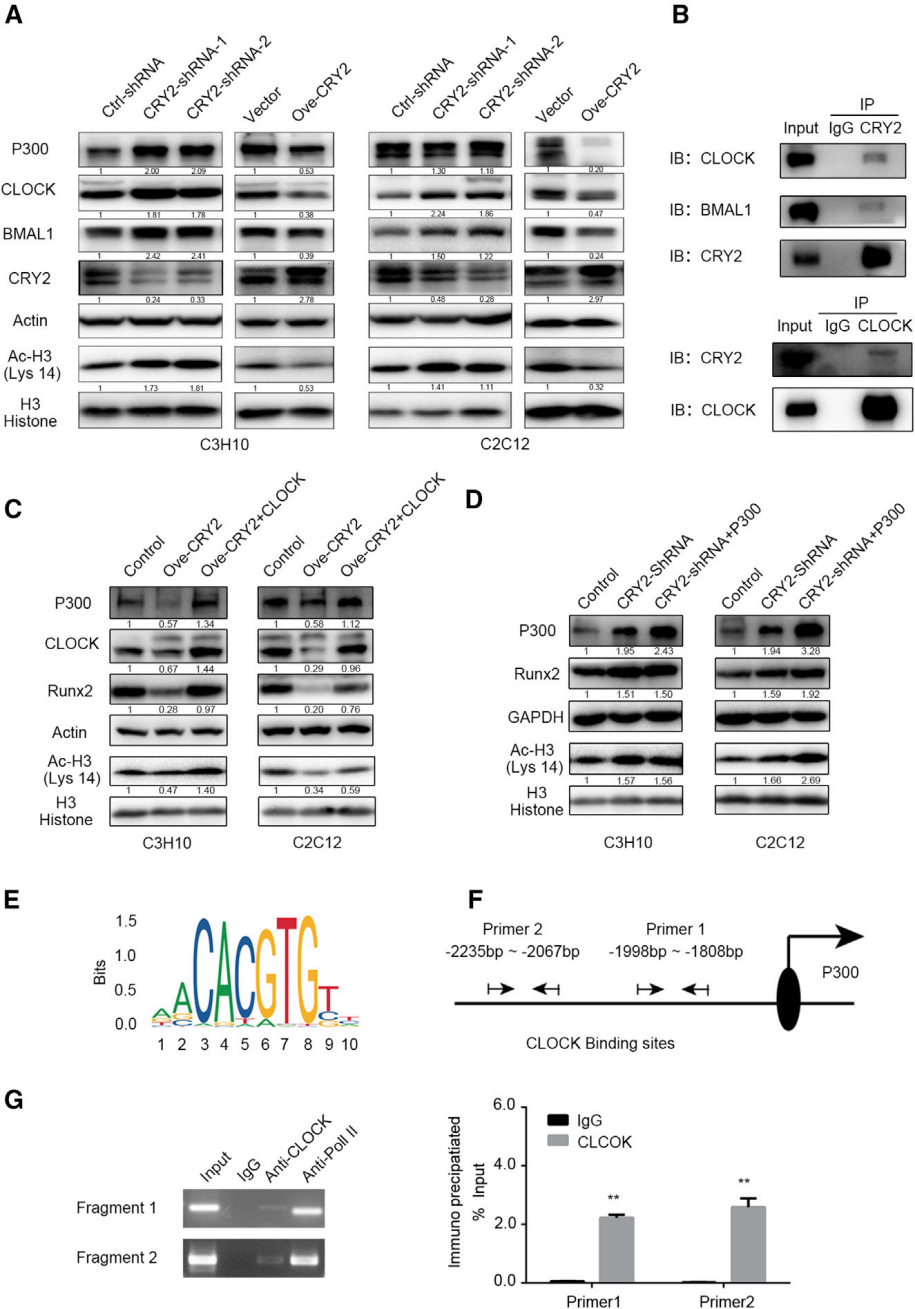
Impact of CRY2 on Osteogenic Differentiation through the Repression of CLOCK/BMAL1/P300 Expression and Histone 3 Acetylation (A) Analysis of CRY2, CLOCK, BMAL1, P300, and acetyl-histone 3 (Lys 14) expression in CRY2 overexpressing and silencing cell lines. (B) Coimmunoprecipitation results showing interaction of CRY2 with CLOCK and BMAL1 in mesenchymal stem cells (MSCs). (C) Detection of P300, CLOCK, Runx2, acetyl-H3 (Lys 14), and H3 histone in C3H10 and C2C12 cell lines with or without overexpression of CRY2 and/or CLOCK. (D) Detection of P300, Runx2, acetyl-H3 (Lys 14), and H3 histone in C3H10 and C2C12 cell lines with or without silencing of CRY2 and/or overexpression of P300. (E) Transcription start site (TSS) binding motives for CLOCK obtained from JASPAR. (F) The promoter region of P300 containing the putative CLOCK binding sites. (G) Binding sites of CLOCK to the promoter regions of P300 detected by ChIP. IgG, negative control; Input, starting sonicated DNA; Pol II, RNA polymerase II positive control. β-actin and GAPDH are a protein-loading control. All assays were repeated at least three times. The data are the mean ± SD (n = 3). n.s., not significant, *p < 0.05, **p < 0.01, ***p < 0.001.

To investigate further the molecular mechanism of osteogenic differentiation regulated by CRY2, we focused on the Cry repressor complex and CLOCK/BMAL1 complex in response to osteogenic stimulation in CRY2 overexpression and silencing cells. As shown in Figure 5A, the expression of CRY2 was significantly reduced in shRNA-expressing cells and increased in cDNA-transfected cells compared with control cells, whereas CLOCK and BMAL1 expression levels were increased in CRY2 shRNA-transduced cells and reduced in CRY2-overexpression cells compared with controls (Figure 5A). Thus, we hypothesized that the CLOCK/BMAL1 complex may mainly participate in the CRY2-mediated osteogenic differentiation. Therefore, we performed coimmunoprecipitation experiments and revealed that CRY2 could bind to the CLOCK/BMAL1 complex in MSCs (Figure 5B). Previous reports have validated that bone morphogenetic protein 2 (BMP-2) regulates osteoblast differentiation through increasing Runx2 acetylation by P300.^19^ To investigate further the molecular mechanism of the CLOCK/BMAL1 complex in osteogenic differentiation induced by CRY2, we tested the expression of P300 and acetyl-histone H3 (Lys 14) in CRY2 silencing and overexpression cells. The results showed that the expression of P300 and acetyl-histone H3 (Lys 14) was upregulated after CRY2 silencing. On the contrary, when CRY2 was overexpressed, the expression of P300 and acetyl-histone H3 (Lys 14) was remarkably suppressed (Figure 5A). Given that the dimer of CLOCK/BMAL1 could regulate the expression of P300, we ectopically expressed CLOCK after overexpression of CRY2, indicating that expression of P300 and Runx2 was markedly increased (Figure 5C) and that overexpression of P300 in CRY2 silencing cells could further increase the Runx2 expression (Figure 5D). To assess further whether the CLOCK/BMAL1 complex could directly participate in the transcriptional regulation of P300, we used bioinformatics and found that the sequence from −1,838 bp to −1,850 bp (5′-TGATCCGCCCTC-3′) and from −2,134 bp to −2,146 bp (5′-CCGCCCGCCTTG-3′), located at the promoter region of P300, might bind to CLOCK (Figures 5E and 5F). Therefore, we performed ChIP assay and found that CLOCK directly bound to the promoter region of P300 to promote the transcription of P300 (Figure 5G). These results unraveled a mechanism that knockdown of CRY2 promotes osteogenic differentiation through the CLOCK/BMAL1-mediated upregulation of P300, which induces histone H3 acetylation and promotes transcriptional activity of Runx2.

### Detection of CRY2, miR-7-5p, STAT3, and P300 in Human MSCs

**Figure 6:**
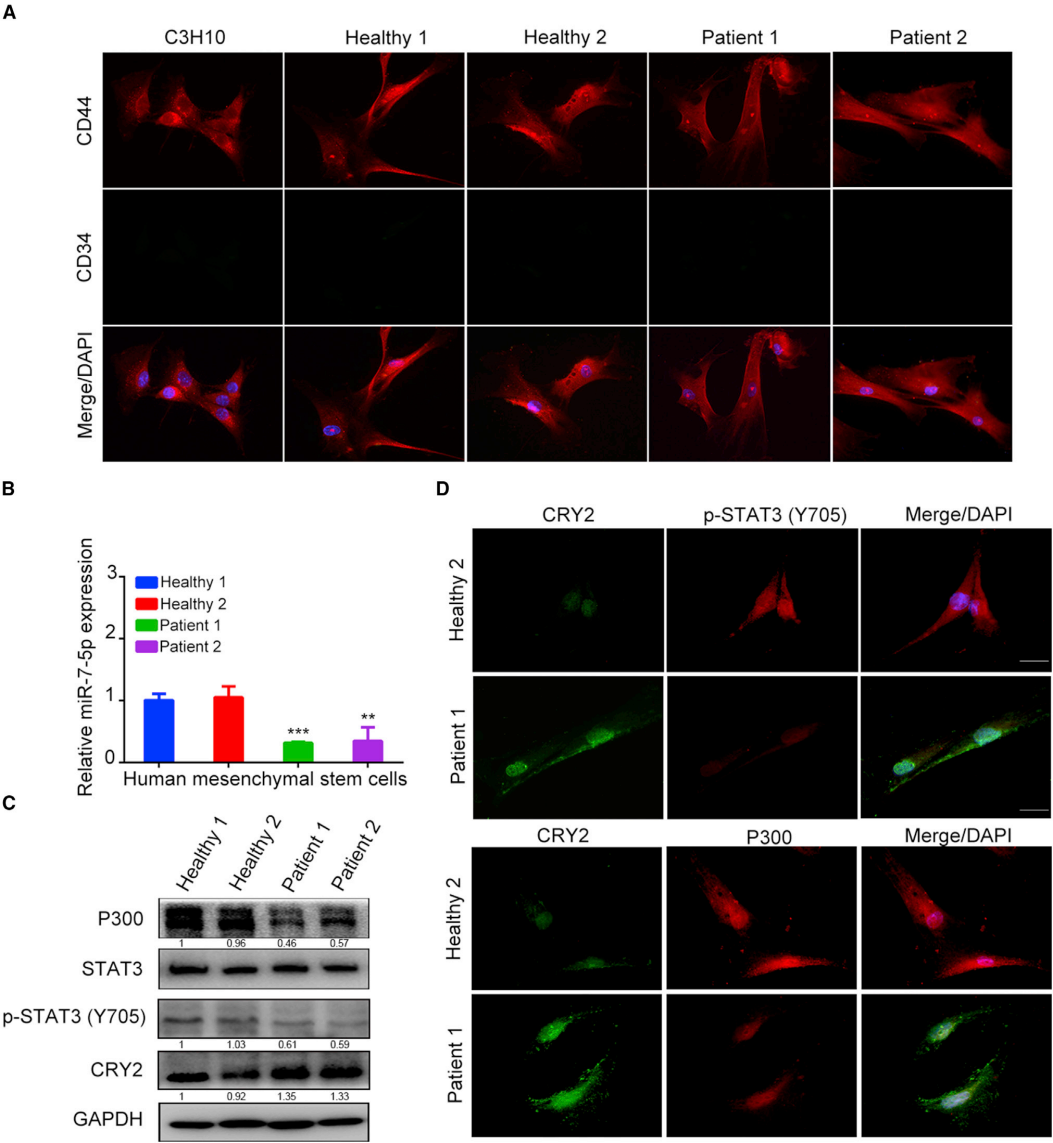
Detection of CRY2, miR-7-5p, STAT3, and P300 in Human MSCs (A) CD34 and CD44 were detected in human bone marrow-derived MSCs by immunofluorescence staining, and C3H10 was used as positive control. (B) Quantitative real-time PCR analysis of miR-7-5p expression in osteoporotic and healthy MSCs. U6 was used as an internal control. (C) Western blot analysis of STAT3, P300, and CRY2 expression in osteoporotic and normal MSCs. (D) Expression of p-STAT3, CRY2, and P300 in osteoporotic and healthy MSCs was analyzed by immunofluorescent staining. GAPDH is a protein-loading control. All assays were repeated at least three times. The data are the mean ± SD (n = 3). n.s., not significant, *p < 0.05, **p < 0.01, ***p < 0.001.

To validate the above findings, human MSCs isolated from bone marrow of 2 osteoporosis and 2 healthy human subjects were detected for the expression levels of mi-7-5p, CRY2, p-STAT3, and P300. Following 2 passages, immunofluorescent staining was performed to detect the expression of MSC marker CD44, whereas the hematopoietic cell marker CD34 was tested as a negative control, and mouse MSCs were used as a positive control (Figure 6A). Further tests showed that the expression of miR-7-5p was lower in osteoporotic patients than in bone healthy subjects (Figure 6B). The expression of CRY2 was much higher in osteoporotic patients than in bone healthy subjects, but P300 and p-STAT3 were lower in osteoporotic MSCs than in healthy MSCs (Figures 6C and 6D). All of these results suggest that the relationship among CRY2, miR-7-5p, p-STAT3, and P300 likely exists in human MSCs, which may indicate a potential clinical application for a treatment strategy of osteoporosis.

## Discussion

In the present study, we have identified an uncharacterized signal pathway in which CRY2 is suppressed by miR-7-5p to promote bone formation. We show that CRY2 negatively regulates the expression of Runx2, ALP, OCN, and OPN to repress osteoblast differentiation. However, CRY2 can be directly targeted by miR-7-5p, which is transactivated by STAT3 to induce osteoblast differentiation. Reintroduction of CRY2 abolishes the osteogenic differentiation induced by miR-7-5p overexpression. CRY2 is able to bind to the CLOCK/BMAL1 complex to suppress the function of CLOCK/BMAL1, whereas interruption of CRY2 activates the function of CLOCK/BMAL1 and the transcription of P300 to promote the acetylation of histone 3 and the expression Runx2, which eventually enhances osteogenesis.

Our previous study has shown that the SNP rs2292910 of *Cry2* is associated with osteoporosis in a Chinese geriatric cohort,^20^ because the SNP rs2292910 is located at the 3′ UTR region of CRY2, which may affect bone formation as a flanking sequence to be targeted by miR-7-5p. However, a similar SNP site was not found or predicted in the 3′ UTR region of mouse CRY2 mRNA. Thus, whether the SNP rs2292910, which we previously reported, is associated with the targeted regulation of CRY2 by miR-7-5p may still need more investigation. Hence, we focused on the role of CRY2 in osteogenic differentiation. A study reported that the mice lacking CRY2 displayed significantly higher bone volume,^7^ and another study revealed that the mice lacking *cry1* and *cry2* had an increase of bone-formation parameters (mineral apposition rate and bone-formation rate).^5^ Thus, we mainly investigated the correlation between miRNA and CRY2 mRNA during osteoblast differentiation. Bioinformatic analysis showed that miR-7-5p might directly target the 3′ UTR of CRY2 (Figure 2A), which was confirmed by the luciferase reporter assay (Figures 2E and 2F).

A previous study reported that miR-7-5p may directly target the 3′ UTR of nuclear factor (NF)-κB p65 to regulate negatively its expression.^21^ Because, the NF-κB pathway positively regulates the binding of receptor activator of NF-κΒ ligand (RANKL) to RANK to suppress osteoblast differentiation,^22^ miR-7-5p may play a decisive role in the maintenance of bone metabolism balance. Beyond that, numerous studies have demonstrated that miR-7-5p may play vital roles in the generation or development of various tumors. miR-7-5p interacts with urothelial carcinoma associated 1 (UCA1) to suppress the degradation of epidermal growth factor receptor (EGFR) through binding to the EGFR 3′ UTR.^23^ miR-7-5p functions as a tumor suppressor to inhibit cancer cell growth, invasion, and metastasis in breast cancer,^24^, ^25^, ^26^ glioma,^27^ bronchial epithelial cells,^28^ neuroendocrine neoplasm,^29^ liver cancer,^30^ and intestinal epithelial cells.^30^ However, miR-7-5p also promotes tumorigenesis in colorectal cancer cells.^31^ Our study reported a novel function of miR-7-5p that is to regulate positively the osteogenesis through the downregulation of CRY2. Moreover, overexpression of miR-7-5p resulted in an increase of mineral nodules and the upregulation of osteoblast marker genes, such as ALP, Runx2, OCN, and type I collagen (Figure 3). To validate the effects of CRY2 downregulation on osteogenesis induced by miR-7-5p overexpression, we silenced the CRY2 expression. We found that silencing of CRY2 led to the increase of osteogenic differentiation and the formation of mineral nodules, as indicated by the high expression of osteogenic gene markers (Figure 1).

In addition, studies have shown that CRY2 functions as a core component of the circadian clock to regulate gene expression negatively.^31^ At the molecular level, circadian clocks are regulated by the transcription-translation feedback loop. Besides of the circadian element, CLOCK, BMAL1, period (per1 and per2), and cryptochrome (cry1 and cry2) also participated in circadian clocks.^32^, ^33^, ^34^ CRY2 regulates the circadian cycle through the repression of the CLOCK/BMAL1-mediated transcription of E-box genes. Previous studies have revealed that circadian clock genes could regulate fibroblast-like synoviocytes and chondrocyte development.^35^ Moreover, CRY suppresses BMAL1-Ser90 phosphorylation through binding to *CK2β* to regulate negatively the mammalian circadian clock.^36^ The circadian transcriptional factors promote the calvarial bone formation.^37^ BMP-2 or extracellular signal-regulated kinase (Erk) signaling stimulates the acetylation of H3 histone by P300 to promote osteoblast differentiation and bone formation.^19^^,^^38^^,^^39^ Hence, we tested the relationship between CLOCK/BMAL1 and P300 during osteoblast differentiation and bone formation. Our results suggest that CLOCK functions as a transcription factor to induce the transcription of P300. We also found that the expression of CRY2 was upregulated in MSCs derived from osteoporotic patients, but the expression of miR-7-5p, p-STAT3, and P300 was decreased in these cells (Figure 6).

**Figure 7:**
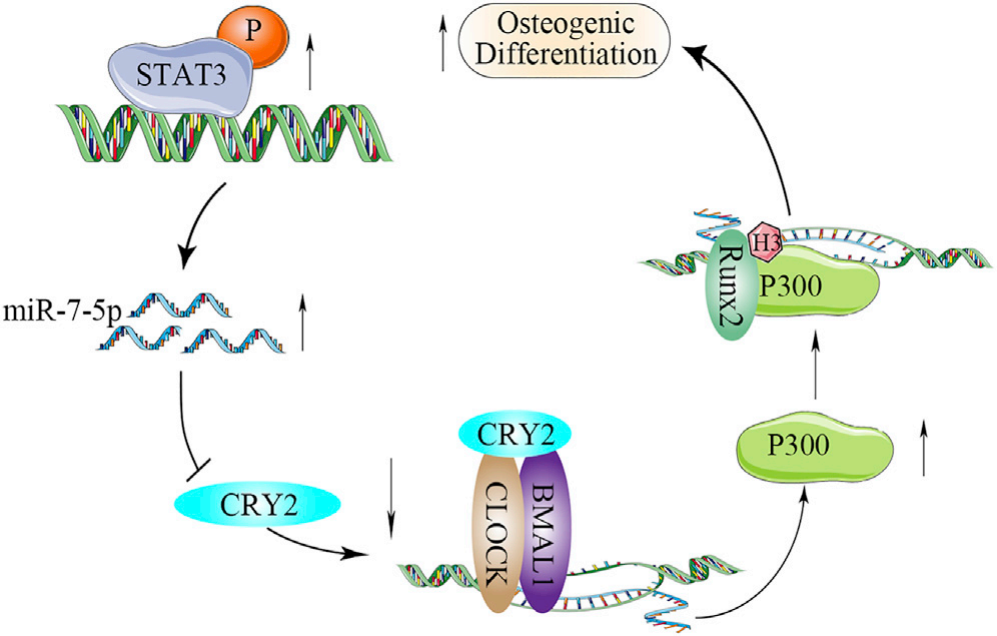
A Schematic Model Showing That miR-7-5p, Transactivated by STAT3, Promotes Osteoblast Differentiation through the CRY2-Mediated Suppression of CLOCK/BMAL1/P300 Expression and Histone 3 Acetylation

Taken together, our data demonstrate that CRY2 is suppressed by miR-7-5p during osteogenesis. The phosphorylation of STAT3 directly regulates miR-7-5p expression, and miR-7-5p induces osteogenic differentiation through repression of CRY2, which inhibits the expression of the CLOCK/BMAL1 complex. Furthermore, CLOCK/BMAL1 mediates the transcription of P300 through binding to the promoter region of the P300 E-box to promote the formation of a transcriptional complex with Runx2, which enhances the acetylation of H3 histone for the progression of osteogenic differentiation (Figure 7). Therefore, a distinct signaling, including STAT3/miR-7-5p/CRY2, has been revealed during osteoblast differentiation, which may provide a potential therapeutic strategy against disorders associated with osteoporosis.

## Materials and Methods

### Cell Culture and Differentiation

Cell lines were purchased from ATCC (Manassas, VA, USA), including C3H10, C2C12, and 293T cells, and cultured in DMEM, supplemented with 10% fetal bovine serum (FBS). Osteoblast differentiation was induced with osteogenic medium containing 50 μg/mL ascorbic acid, 10 mM β-glycerophosphate, and 1 mM dexamethasone (osteogenic culture conditions), and fresh medium was replaced every 2 days.^40^ Cells were incubated in a humidified atmosphere at 37°C with 5% CO_2_.

### Plasmid Construction

miRNA and anti-miRNA precursor clones of miR-7-5p and anti-miR-7-5p in lentiviral vectors and the lentiviral plasmids containing CLOCK and P300 cDNAs were purchased from LncBio (Shanghai). The lentivirus system for shRNA expression includes three plasmids (pLKO.1-puro, psPAX2, and pMD2.G). The lentiviral vector pCDH-CMV-MCS-EF1-puro was obtained from Addgene System Biosciences (SBI). To knock down CYR2 expression, two shRNAs (CRY2 shRNA-1, 5′-GCTGAATTCGCGTCTGTTTGT-3′ and shRNA-2, 5′-GGATGAATGCCAATTCCTTAC-3′) targeting the mouse CRY2 mRNA sequence were inserted into the lentiviral vector pLKO.1-puro, according to the previously reported methods.^41^ A full-length coding DNA sequence of mouse CRY2 was amplified using the forward primer 5′-GCTCTAGAATGGCGGCGGCTGCTGTGGTGGCAG-3′ and the reverse primer with hemagglutinin (HA) tag: 5′-CAAGGAAAAAAGCGGCCGCTCAAGCGTAATCTGGAACATCGTATGGGTAGGAGTCCTTGCTTGCTGGCTCT-3′. The PCR product was cloned into the lentiviral vector pCDH-CMV-MCS-EF1-puro.

### Western Blot

Cells were lysed using the radioimmunoprecipitation assay (RIPA) lysis buffer (Beyotime, China) containing the protease inhibitor cocktail (Sigma). The protein concentration was determined using the bicinchoninic acid (BCA) Protein Assay Kit (Beyotime). Equal amounts of proteins were separated by 10% SDS-PAGE and transferred to the polyvinylidene fluoride (PVDF) membranes. After blocking, membrane-bound proteins were then incubated overnight at 4°C with primary antibodies. After washing and incubating with secondary antibodies, protein levels were detected using the enhanced chemiluminescence reagents (Millipore). The primary antibody to CRY2 was from Proteintech (USA) (dilution 1:1,000). The antibodies to Runx2, p-STAT3 (Y705), total-STAT3, BMAL1, histone H3, and acetyl-histone H3 (Lys14) were from Cell Signaling Technology (USA) (dilution 1:1,000). The antibodies to p-STAT3 (S727) and P300 were from Santa Cruz (USA) (dilution 1:500). The antibodies to OPN and CLOCK were from Abcam (UK) (diluted 1:1,000). The antibody against beta-actin was from Sigma-Aldrich (USA) (1:10,000). All experiments were repeated three times. All blots were exposed for visualization between 5 s and 2 min. The intensity of protein bands was quantified by ImageJ software (http://imagej.nih.gov/ij/download.html) to calculate the ratios of IntDen (proteins)/IntDen (β-actin or glyceraldehyde 3-phosphate dehydrogenase [GAPDH]) to ensure that the detection of protein bands was linearized.

### RNA Isolation and Real-Time PCR Analysis

Total RNA was extracted from cultured cells using the TRIzol reagent (Invitrogen), according to the manufacturer’s instruction. Real-time PCR was performed on the Applied Biosystems 7500 Real-Time PCR Systems (Applied Biosystems). To detect the miRNA levels, total RNA was reversely transcribed into cDNA using TransScript miRNA for the First-Strand cDNA Synthesis SuperMix kit (TransGen Biotech, China), and real-time-PCR analysis was performed using FastStart Universal SYBR Green Master (Rox) (Roche). U6 was used as an internal control for the miRNA assay, and the reactions were run in triplicates. Relative mRNA or miRNA expression levels were calculated using the 2^−ΔΔCt^ method to determine the fold change in expression between the experimental and control groups.

### Luciferase Reporter Assay

The target gene was predicted by scanning the database. The wild-type 3′ UTR sequence of CRY2 mRNA containing the putative binding site for miR-7-5p was amplified by PCR and cloned into the downstream of the firefly luciferase pGL3 reporter plasmid (Promega, USA). In addition, the mutant 3′ UTR of CRY2 was constructed using the Mut Express MultiS Fast Mutagenesis Kit (Vazyme, China). The mutation primers used were as follows: forward 5′-ACTAAAGCAAGGCGTACTTCCACCCTGTGGCCTGCACTT-3′ and reverse: 5′-GGTGGAAGTACGCCTTGCTTTAGTTGGCCACCGTCGTCC-3′ and forward: 5′-ACTAAAGCAACGGGAAGTTCCACCCTGTGGCCTGCACTT-3′ and reverse: 5′-GGTGGAACTTCCCGTTGCTTTAGTTGGCCACCGTCGTCC-3′. To perform the luciferase assay, HEK293T or C3H10 cells were seeded into 24-well plates with 50% density and cotransfected with WT or mutant plasmid pGL3, pRL-TK Renilla, and miR-7-5p plasmid (or NC) using Lipofectamine 2000. After 48 h, luciferase activity was analyzed using the Dual-Luciferase Reporter System (Promega, USA), according to the manufacturer’s instructions. Firefly luciferase activity was normalized with Renilla luciferase activity.

### ChIP

ChIP was performed using the EZ-ChIP kit (Millipore), according to the manufacturer’s instructions. An anti-mouse p-STAT3 antibody (CST), anti-immunoglobulin G (IgG) control antibodies (1 μg) and positive control anti-RNA Polymerase II antibody (1 μg) were used for the immunoprecipitation. Immunoselected genomic DNA was then used in standard PCR and real-time PCR, as previously described.^42^ The primers used for amplification of the miR-7-5p gene promoter were as follows: fragment 1, forward 5′-AAGGTGGTCAGCGGATTA-3′ and reverse 5′-CCCCAAAAGGTTGAGACA-3′; fragment 2, forward 5′-CCTGGGCAACATAGCAAG-3′ and reverse 5′-AGGTCCACCAAGATCACC-3′; fragment 3, forward 5′-ACAAGTAACTATTCCCAAAG-3′ and reverse 5′-AGCTCACTAAAGCCCATT-3′. The primers used for amplification of the P300 gene promoter were as follows: fragment 1, forward 5′-CACCGTGCCCGGCTAATT-3′ and reverse 5′-GGTGGCTCACGCCTGTAA-3′; fragment 2, forward 5′-TATCCCTGAAGGCCAATC-3′ and reverse 5′-AGGCTGAGGCAGGAGAAT-3′.

### Alizarin Red Staining and Alkaline Phosphatase Staining

Osteogenic induction of cells may generate the formation of mineralized nodules and calcium deposits that could be well determined by Alizarin red staining, according to the literature,^43^ so we performed Alizarin red staining in our study. Specifically, cells were fixed with 4% paraformaldehyde and rinsed three times with one times PBS to remove paraformaldehyde completely. Then the cells were stained with 40 mM alizarin red stain solution (pH 4.2) for 1 h at 37°C to label the calcium deposits. After that, all of the plates were rinsed with distilled water to wash unconjugated Alizarin red and scanned. The alkaline phosphatase staining was performed using the Leagene Alkaline Phosphatase Kit (Leagene; China). Cells were rinsed in PBS and fixed with 4% paraformaldehyde for 30 min at room temperature, washed with PBS, and then stained following the manufacturer’s instruction. The stained cells in plates were photographed. To quantify the Alizarin red-S (AR-S) staining, stained cells were dissolved with 10% (w/v) cetylpyridinium chloride, and the extracted solution was measured by the absorbance at 562 nm.

### ALP Activity Assay

After osteogenic induction, cells were detected for the ALP activity by using a commercial kit (Sigma), according to the manufacturer’s instructions. A bicinchoninic acid (BCA) method (Pierce, Rockford, IL, USA) was performed to detect the protein concentration. Following standard protocols, the relative ALP activity was then normalized to total protein concentration. To quantify the ALP staining, stained cells were dissolved with 10% (w/v) cetylpyridinium chloride, and the extracted solution was measured by the absorbance at 562 nm.

### Isolation of Human Mesenchymal Stem Cells from Bone Marrow of Osteoporosis Patients

Ethical approval for the use of patient samples in this study was granted by the Medical Ethics Committee of the Fifth People’s Hospital of Shanghai City (2014EC (040)). Mesenchymal stem cells were isolated from bone marrow of osteoporotic patients and normal patients (Fifth People’s Hospital of Shanghai Fudan University). Cells were flushed with DMEM and resuspended in DMEM, supplemented with 20% heat-inactivated FBS and 1% penicillin/streptomycin after 800 rpm centrifugation. These cells were cultured for about 14 days with media changes every 2–3 days and used to detect the expression of relative protein by western blot and immunofluorescence.

### Immunofluorescence Staining

Cells were seed in 24-well plates containing cover slides and incubated overnight. The cover slides were washed with PBS and fixed with 4% formaldehyde for 15 min after washing with PBS. Triton X-100 (0.05%) was treated to permeabilize the cells for 5 min, and slides were blocked with 5% BSA. After incubation with primary antibody, the slides were washed, incubated with a secondary antibody conjugated with Alexa Fluor 594 and Alexa Fluor 488 (Jackson ImmunoResearch), washed again with PBS, and stained with DAPI. The mounted slides were viewed with a fluorescence microscope, and images were captured with a fluorescent microscope camera.

### Statistical Analysis

Statistical analyses were performed using GraphPad Prism 6.0 software. Each experiment was performed in triplicate with all the data expressed as the mean ± SD. Statistical differences used Student’s t test and one-way analysis of variance (ANOVA). Values were considered statistically significant at p < 0.05.

## Author Contributions

Conceptualization, G.Y. and Y.H.; Methodology, Z.T.; Investigation, Z.T., T.X., Y.L., and W.F.; Writing – Original Draft, Z.T. and G.Y.; Writing – Review & Editing, Z.T., G.Y., and Y.H.; Funding Acquisition, Y.H.; Resources, G.Y. and Y.H.; Supervision, G.Y. and Y.H.

## Conflicts of Interest

The authors declare no competing interests.
